# ‘Make Hay While the Sun Shines’—The Potential for Seed Production in Rare Terrestrial Orchids Mown During the Flowering Stage

**DOI:** 10.1002/ece3.71578

**Published:** 2025-06-19

**Authors:** Attila Molnár V.

**Affiliations:** ^1^ HUN‐REN–UD Conservation Biology Research Group, Department of Botany University of Debrecen Debrecen Hungary

**Keywords:** fruit formation, herbarium, mowing, *Orchidaceae*, threatened species, vascular plants

## Abstract

The discovery of two specimens of the rare and endemic Caucasian Lizard Orchid (*Himantoglossum formosum*) in 2018 in northern Azerbaijan — both found in a haystack, in the budding and early flowering stages — provided an opportunity to add these specimens to the natural history collection at the University of Debrecen. Following the standard herbarium preparation procedure (dry pressing), an unexpected event was observed: one specimen surprisingly produced a fruit and a large number of fully developed seeds. Despite the absence of tubers, the plant was able to mature its seeds. This fact suggests that mowed orchid specimens with previously pollinated flowers may be capable of maturing seeds, even in the absence of contact with underground organs (roots and tubers). Moreover, this observation may offer new insights into orchid conservation in mowed grasslands. (1) This finding could explain how some orchid species are able to persist in habitats where mowing coincides with their main flowering period (e.g., May and June in temperate Europe). (2) Our results suggest that seeds from herbarium specimens may be successfully utilised for ex situ conservation, thereby aiding in the reintroduction and reinforcement of endangered orchid populations. (3) Significant populations of orchids, including multiple *Himantoglossum* species, are known to inhabit mowed roadside verges. It is likely that seed production in these regularly mown areas plays a role in the northward expansion of certain species.

## Graphical abstract

The discovery of two specimens of the endemic Himantoglossum formosum in Azerbaijan, both in the budding and early flowering stages, was prepared following the standard herbarium preparation procedure, an unexpected event was observed: one specimen surprisingly produced a fruit and a large number of fully developed seeds. Despite the absence of tubers, the plant was able to mature its seeds using nutrients from the above‐ground organs. This observation may offer new insights into the biology and conservation of orchids in mowed grasslands.

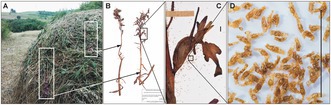

## 1 Introduction

Semi‐natural habitats host many endangered species, yet they are increasingly declining and fragmenting globally (Tilman et al. 2001) due to the intensification of land use (Tscharntke et al. 2005; Firbank et al. 2008) and the erosion of traditional agricultural practices (Bignal and McCracken 1996). Plant species that thrive in semi‐natural grasslands often tolerate, or even prefer, elements of traditional land use, such as grazing and mowing (Eriksson et al. 2015; Pykälä et al. 2005). However, these practices are disappearing with the advancement of modern agricultural technologies.

An analysis of the decline in the distribution ranges of orchids in Estonia and the United Kingdom revealed a greater decline in species found on drier soils, as well as those typical of open habitats. Moreover, the results suggest that grazing and mowing of competing vegetation yield significant benefits for the most vulnerable terrestrial orchid species (Kull and Hutchings 2006). The dramatic decline in populations of many orchid species in recent decades has been attributed to the loss and degradation of their natural and semi‐natural habitats, primarily due to secondary succession following the intensification or abandonment of traditional land use (Schrautzer et al. 2011; Štípková and Kindlmann 2021). Among the key elements of traditional land use in semi‐natural grasslands, mowing stands out. The importance of mowing in the persistence of orchid populations, including species such as *Anacamptis morio*, *Dactylorhiza incarnata* , *D. lapponica* , *Himantoglossum adriaticum*, *Nigritella nigra*, and *Spiranthes spiralis*, has become increasingly evident (Smith and Cross 2016; Schrautzer et al. 2011; Sletvold et al. 2010, 2013; Bódis et al. 2019; Moen and Øien 2002; Paušič et al. 2017). In the era of intensifying and industrializing agricultural practices, replacing traditional manual mowing of species‐rich grasslands remains a major challenge for conservation agencies and organizations. The size, spatial distribution, and timing of mowed areas are all crucial factors.

Currently, the timing of mowing from a conservation perspective is usually determined based on the lifecycle of threatened plant species, typically after their fruits have ripened and seeds have been set. However, for centuries, traditional farmers did not consider the life cycles of individual plant species when deciding when to make hay or graze, instead focusing on practical aspects such as animal preferences, plant nutritional value, yield, and impact on milk production (Molnár 2017). In haymaking, not only does the plant species but also the timing of mowing significantly influence the quality of the fodder. Thus, mowing traditionally occurred before the mass flowering of grasses.

The propagule content of mowed hay holds practical significance for traditional farmers (cf. Babai and Molnár 2014), and hay can also be used for ecological restoration purposes (Török et al. 2012). However, assessing the species composition of hayseed can be challenging (Kiehl et al. 2010), particularly for extremely small‐seeded species such as orchids. Consequently, little is known about the role of hay in dispersing these taxa.

**FIGURE 1:**
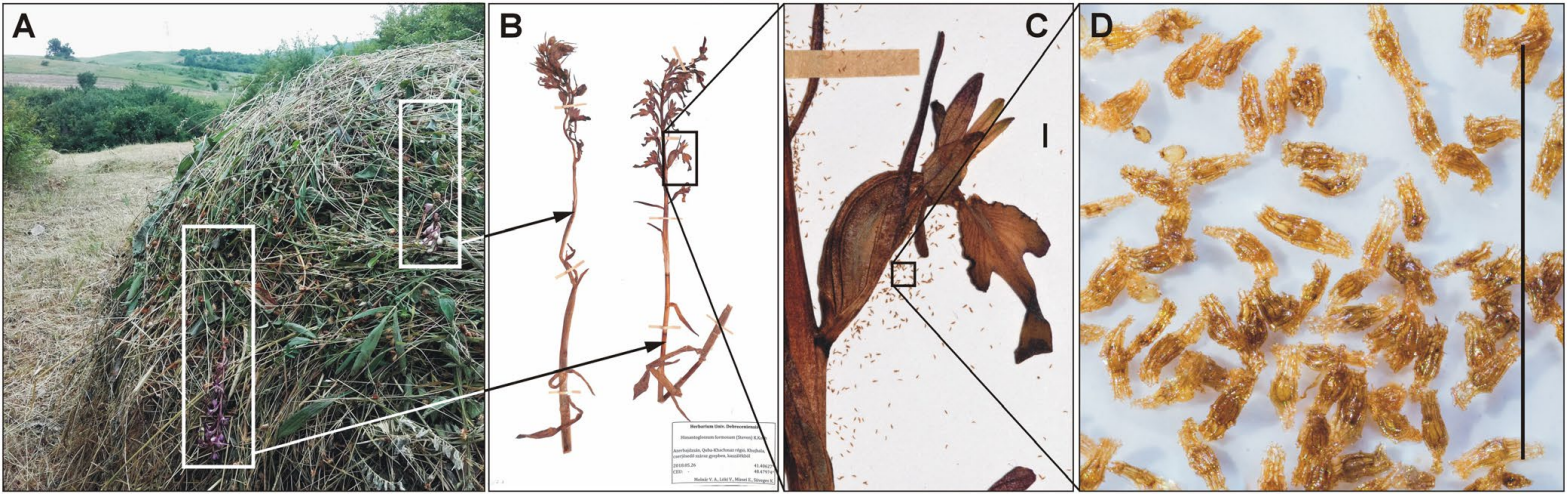
Mowed Caucasian Lizard‐Orchid (*Himantoglossum formosum*) producing viable seeds. (A) Two individuals in haystack, mowed in budding and early flowering phenological stage (Azerbaijan, 26. May 2018), (B) herbarium sheet of mowed specimens, (C) fruit ripened from the dry, pressed specimen with seeds on herbarium sheet, (D) seeds. Scale bars (C, D) represent 5 mm. Photographs (B and C) by A. Molnár V., A and D by V. Löki.

This study was prompted by a fortuitous event: the discovery of two specimens of the rare, endemic terrestrial orchid species *Himantoglossum formosum* (Steven) K. Koch, found in a haystack (Figure 1A), which had been mowed during anthesis. The writing of this article was initiated by the unexpected finding that one of the specimens, discovered after mowing and preserved in a herbarium, surprisingly produced a fruit and ripened seed.

## 2 Materials and Methods

*Himantoglossum formosum* is one of the least known species among temperate Eurasian terrestrial orchids. For nearly 180 years after its description (Steven 1813), little was known about the species, and its extinction was even predicted by European botanists (Baumann and Künkele 1982: 156). The species was rediscovered and photographed for the first time by German orchid enthusiasts in 1994. Since then, a few small populations have been detected. Genetically (Sramkó et al. 2014) and morphologically (Bateman et al. 2017), it is well established as a distinct species. It is listed in the Red Data Book of the Russian Federation (Averyanov 2008) and, as a vulnerable species, in the IUCN Red List of Threatened Plants (Walter and Gillett 1997).

Two freshly mown generative individuals of *H. formosum* were found in a haystack (Figure 1A) near a known significant population, near the village of Hucbala/Khuxvala (N41.40627° E48.47974°, Quba region, Northern Azerbaijan) on 26th May 2018. After undergoing traditional drying and pressing procedures, the specimens were deposited in the herbarium of the Department of Botany, University of Debrecen (DE, Debrecen, Hungary; voucher number: DE‐Soo‐45,975; Figure 1B).

## 3 Results and Discussion

Following the standard herbarium preparation procedure (dry pressing), an unexpected event was observed: one of the orchid individuals mown during the flowering stage produced a capsule (Figure 1C), formed from a flower that had been pollinated before mowing but matured afterward. The fruit contained a large quantity of mature seeds (Figure 1C), which showed normally developed embryos (Figure 1D).

We have demonstrated that a specimen of *Himantoglossum formosum* mown during its flowering stage can produce seeds after being physically isolated from its tuber. This finding may provide new insights into orchid conservation in mowed grasslands.

1. This phenomenon may be related to the characteristic reproductive biology and life history of orchids. Since orchid seeds are very small, with a significant portion of their volume occupied by air (Arditti and Ghani 2000), resulting in an extremely low mass. For example, the thousand‐seed mass for *Himantoglossum jankae* and *H. adriaticum* is 0.0006 and 0.0013 g, respectively (Sonkoly et al. 2016). The embryo is undifferentiated and lacks the reserve nutrients required for its initial development (Burgeff 1936). In most cases, mycorrhizal fungal partners are essential for seed germination and the initial differentiation of the seedling (Rasmussen 1995). Twin‐tuber orchids, such as *Himantoglossum* species, possess two tubers: one, the mother‐tuber, formed the previous year and nourished the shoot during that season. However, by the time the plant flowers and the fruits ripen, the mother‐tuber becomes limp and wrinkled, with its reserve nutrients depleted, making it unlikely to support the shoot (and thus the fruits). The other tuber, the daughter‐tuber, is firm and compact and will use the reserve nutrients stored within it to develop the plant's shoots for the following year. Therefore, the tuber is unlikely to play a significant role in seed formation, as evidenced by the fact that only one of the fresh daughter tubers is collected in species harvested for ‘salep’ (a hot winter beverage in the Eastern Mediterranean) (Molnár, Süveges, et al. 2017).
2. This observation may explain how some orchid species can persist in habitats where mowing coincides with their main flowering period (e.g., May and June in temperate Europe). This ability could offer a significant advantage for orchids that colonise regularly mown habitats under heavy anthropogenic influence, such as roadsides (Fekete et al. 2017, 2019, 2020) and cemeteries (Löki et al. 2015, 2019; Molnár, Nagy, et al. 2017; Molnár, Takács, et al. 2017; Molnár V. et al. 2021). The results support the notion that traditional haymaking methods facilitate orchid seed production, even when mowing occurs during the flowering period. These findings align with the recommendations of Molnár and Babai (2021) regarding the application of traditional ecological knowledge in biodiversity conservation.
3. The conservation of certain plant species can be effectively supported by seeds stored in natural history collections (Godefroid et al. 2011; Molnár 2017; Nualart et al. 2017), and some terrestrial orchids are capable of building persistent in situ seed banks (Whigham et al. 2006). Our results suggest that seeds from herbarium specimens may be successfully used for ex situ conservation, facilitating the reintroduction and population reinforcement of endangered orchids. Further research is needed to explore the longevity of orchid seeds stored in natural history collections.
4. Significant populations of several orchid species, including multiple *Himantoglossum* species, are known to inhabit roadside verges (Fekete et al. 2017, 2019, 2020, 2023). It is likely that seed production in these regularly mown stands plays a role in the northward expansion of some species, as documented for H. hircinum (Good 1936; Carey 1999; Pfeifer et al. 2006; van der Meer et al. 2016), *H. adriaticum* (Óvári 2017; Tóth and Teleki 2024; Molnár V. et al. 2024), and *H. calcaratum* (Kacsinecz et al. 2025).

## Author Contributions

**Attila Molnár V.:** conceptualization (lead), data curation (lead), funding acquisition (lead), investigation (lead), project administration (lead), visualization (lead), writing – original draft (lead), writing – review and editing (lead).

## Conflicts of Interest

The author declares no conflicts of interest.

## Data Availability

Data sharing is not applicable to this article as no new data were created or analyzed in this study.
